# The IncP-6 Plasmid p10265-KPC from *Pseudomonas aeruginosa* Carries a Novel ΔIS*Ec33*-Associated *bla*_KPC-2_ Gene Cluster

**DOI:** 10.3389/fmicb.2016.00310

**Published:** 2016-03-10

**Authors:** Xiaotian Dai, Dongsheng Zhou, Wei Xiong, Jiao Feng, Wenbo Luo, Guangming Luo, Haijing Wang, Fengjun Sun, Xiangdong Zhou

**Affiliations:** ^1^Department of Pulmonology, Southwest Hospital, Third Military Medical UniversityChongqing, China; ^2^State Key Laboratory of Pathogen and Biosecurity, Beijing Institute of Microbiology and EpidemiologyBeijing, China; ^3^Department of Pharmacy, Southwest Hospital, Third Military Medical UniversityChongqing, China

**Keywords:** *Pseudomonas aeruginosa*, KPC-2, p10265-KPC, IncP-6

## Abstract

*Pseudomonas aeruginosa* strain 10265 was recovered from a patient with pneumonia in a Chinese public hospital, and it displays the carbapenem resistance phenotype due to the acquisition of a non-conjugative but mobilizable IncP-6-type plasmid p10265-KPC. p10265-KPC carries a Tn*5563*-borne defective *mer* locus, and a novel ΔIS*Ec33*-associated *bla*_KPC-2_ gene cluster without paired inverted repeats and paired direct repeats at both ends. Mobilization of this ΔIS*Ec33*-associated element in p10265-KPC would be attributed to homologous recombination-based insertion of a foreign structure Tn*3-*IS*Apu1*-*orf7*-IS*Apu2*- IS*Kpn27-*Δ*bla*_TEM-1_*-bla*_KPC-2_*-*ΔIS*Kpn6- korC-orf6*-*klcA-*Δ*repB* into a pre-existent intact IS*Ec33*, making IS*Ec33* truncated at the 3′ end. The previously reported pCOL-1 represents the first sequenced KPC-producing IncP-6 plasmid, while p10265-KPC is the second one. These two plasmids carry two distinct *bla*_KPC-2_ gene clusters, which are inserted into the different sites of the IncP-6 backbone and have different evolutionary histories of assembly and mobilization. This is the first report of identification of the IncP-6-type resistance plasmid in China.

## Introduction

*Klebsiella pneumoniae* carbapenamases (KPCs) were initially discovered in USA in 1996, and they have disseminated worldwide among Enterobacteriaceae, *Pseudomonas* and *Acinetobacter* with *K. pneumoniae* being the most predominate species (Munoz-Price et al., 2013; Chen et al., 2014b). At least 23 KPC protein variants (KPC-2 to KPC-24; KPC-1 is essentially identical to KPC-2) have been identified^1^. The *bla*_KPC_ genes are typically present on plasmids, varying in size, genetic structure and incompatibility group (e.g., IncFII, FIA, I2, A/C, N, X, R, P, U, W, L/M, and ColE; Munoz-Price et al., 2013; Chen et al., 2014b). Chromosomal location of *bla*_KPC_ has also been evidenced in *Pseudomonas aeruginosa*, indicating that the *bla*_KPC_ genes can be integrated into host genome (Villegas et al., 2007; Cuzon et al., 2011). KPC-producing bacteria are resistant to almost all β-lactams and often to other classes of antibiotics (such as aminoglycosides, quinolones, and tetracyclines), resulting from the co-selection of additional resistance determinants encoded by insertion sequence-, integron- and transposon-associated mobile gene clusters and, thereby, leaves few or no antimicrobial treatment options (Munoz-Price et al., 2013; Chen et al., 2014b).

At least thirteen incompatibility groups, IncP-1 to IncP-7 and IncP-9 to IncP-14, have been recognized for the plasmids found in the natural isolates of *Pseudomonas*, and about one third of these plasmids characterized belong to the IncP-2 group which typically have a size > 300 kb (Sagai et al., 1976; Boronin, 1992). Plasmids belonging to IncP-2, IncP-5, IncP-7, IncP-10, IncP-12, and IncP-13 incompatibility groups have a narrow host range and cannot be transferred from *Pseudomonas* to *Escherichia coli*, while the other IncP types especially including IncP-1, IncP-4, and IncP-6 seem to have a broad host range (Sagai et al., 1976; Boronin, 1992; Xiong et al., 2013). IncP-1 corresponds to IncP in the *E. coli* plasmid classification system, and plasmids of this group can transfer and replicate virtually in all Gram-negative bacteria, contributing to the spread of antibiotic and heavy metal resistance (Popowska and Krawczyk-Balska, 2013).

The IncP-6 plasmids are capable of replicating in both *E. coli* (where there are assigned into the IncG group) and *Pseudomonas*. Only a few IncP-6 plasmids, such as Rms149 from *P. aeruginosa* (Haines et al., 2005), pCOL-1 from *P. aeruginosa* (Naas et al., 2013) and pRIO-5 from *Serratia marcescens* (Bonnin et al., 2012), have been fully sequenced, and all these plasmid have acquired various mobile genetic structures harboring resistance markers. In addition, pRSB105 from an uncultured eubacterium represents a mosaic plasmid that carries the IncP-6 backbone as well as the Rep1 replicon module, most likely contributing to the extension of plasmid’s host range (Schluter et al., 2007).

Data presented here reveal that *P. aeruginosa* strain 10265 harbors a novel IncP-6 resistance plasmid p10265-KPC. The complete sequence of p10265-KPC was determined and compared with other sequenced IncP-6 plasmids. p10265-KPC carries a novel ΔIS*Ec33*-associated *bla*_KPC-2_ gene cluster as well as a Tn*5563*-borne defective mercury resistance (*mer*) gene locus, providing further insights into drug resistance mechanism of the KPC-encoding IncP-6 plasmids.

## Materials and Methods

Bacterial species was identified using Bruker MALDI Biotyper (Bruker Daltonics, Bremen, Germany) and 16S rRNA gene sequencing (Frank et al., 2008). The major acquired carbapenemase and extended-spectrum β-lactamase genes were detected by PCR, followed by amplicon sequencing on ABI 3730 Sequencer (Applied Biosystems, Foster City, CA, USA; Chen et al., 2015). The experimental protocols were approved by the Ethics Committee of the Third Military Medical University.

Plasmid electroporation or conjugal transfer was performed with *E. coli* TOP10 (LacZ^-^, resistant to streptomycin and tetracycline) and EC600 (LacZ^-^, resistant to nalidixic acid and rifampicin) being used as recipient for selection of *bla*_KPC_-positive electroporants or transconjugants, respectively (Chen et al., 2015). Transfer of the *bla*_KPC_ gene on the plasmid was determined by S1-PFGE and Southern blot hybridization (Lee et al., 2012; Chen et al., 2015).

Activity of Ambler class A/B/D carbapenemases in bacterial cell extracts was determined by CarbaNP test (Dortet et al., 2012) with modifications (Chen et al., 2015). Bacterial antimicrobial susceptibility was tested by VITEK 2 (BioMérieux Vitek, Hazelwood, MO, USA) and interpreted as per Clinical and Laboratory Standards Institute guidelines (Twenty-Fourth Informational Supplement M10-S24, 2014).

Plasmid DNA was isolated from *E. coli* electroporant using Qiagen large construct kit (Qiagen, Hilden, Germany), and sequenced by whole-genome shotgun strategy in combination with Illumina HiSeq 2500 (Illumina, San Diego, CA, USA) sequencing technology. The contigs were assembled with Velvet, and the gaps were filled through combinatorial PCR and Sanger sequencing on ABI 3730 Sequencer. The genes were predicted with GeneMarkS^TM^ and further annotated by BLASTP and BLASTN against UniProt and NR databases. Gene organization diagrams were drawn with Inkscape^2^. The complete sequence of p10265-KPC was submitted to GenBank under accession number KU578314.

## Results and Discussion

### Case Report

In September 2010, an 81-year-old male with hemafecia visited a public hospital in Beijing of China, and the progressive symptoms of fever, couch, and pulmonary infection were observed after hospitalization. The patient had the underlying diseases hypertension, diabetes, multiple cerebral infarction, and chronic renal insufficiency. The patient received long-term hospital care under the hospital and was bed-ridden with indwelling catheter. His symptoms of hemafecia were generally well controlled during hospitalization, but he started to suffer from the recurrent urinary tract infections since August 2013. About 2 weeks later, bacterial colonies were observed after cultivation of the urine specimens on the Mueller-Hinton agar, and the bacterial isolate designated 10265 was identified as *P. aeruginosa*. Based on the antimicrobial susceptibility test results, the patient received the intravenous administration with amikacin, and his symptoms associated with urinary tract infections progressively disappeared.

### Overview of Plasmid p10265-KPC

Screening for the *bla*_GES_, *bla*_KPC_, *bla*_SME_, *bla*_IMI_, *bla*_BIC_, *bla*_IMP_, *bla*_V IM_, *bla*_NDM_, *bla*_TMB_, *bla*_FIM_, *bla*_SPM_, *bla*_DIM_, *bla*_GIM_, *bla*_SIM_, *bla*_AIM_, *bla*_SMB_, *bla*_OXA_, *bla*_CTX-M_, *bla*_TEM_, *bla*_SHV_, *bla*_GES_, *bla*_PER_, *bla*_V EB_, and *bla*_OXA_ genes by PCR (Chen et al., 2015) indicated the presence of only *bla*_KPC-2_ (but not any of the other *bla* genes tested) in strain 10265. Electroporation of the plasmid DNA of strain 10265 into *E. coli* TOP10 generated a *bla*_KPC_-positive electroporant 10265-KPC-TOP10, but repeated attempts of plasmid conjugal transfer with *E. coli* EC600 being used as recipient and strain 10265 as donor failed to obtain a *bla*_KPC_-positive *E. coli* transconjugant. S1-PFGE followed by Southern hybridization (Chen et al., 2015) indicated the presence of a ∼40 kb plasmid, being able to hybridize with a *bla*_KPC_-specific probe (Lee et al., 2012), in both 10265 and 10265-KPC-TOP10 (data not shown)^.^10265 and 10265-KPC-TOP10 but not TOP10 have the class A carbapenemase activity (data not shown). Strain 10265 is resistant to all the β-lactam, β-lactamase inhibitor, fluoroquinolone, and sulfonamide drugs tested but remains susceptible to aminoglycosides, while 10265-KPC-TOP10 is resistant to β-lactams and β-lactamase inhibitors but remains susceptible to all the other drugs (**Table 1**). Taken together, strain 10265 harbors a non-conjugative plasmid p10265-KPC, which carries the *bla*_KPC-2_ gene to mediate the resistance to β-lactams including monobactam and carbapenems.

**Table 1:** Antimicrobial resistance phenotypes of *Pseudomonas aeruginosa* and *Escherichia coli* with plasmid p10265-KPC.

Antibiotics	MIC (mg/L)/antimicrobial susceptibility		
	*P. aeruginosa* 10265	*E. coli* 10265-KPC-TOP10	*E. coli* TOP10
**Penicillins**			
Ampicillin	≥32/R	≥32/R	4/S
Ampicillin/sulbactam	≥32/R	≥32/R	4/S
Piperacillin	≥128/R	≥128/R	≤4/S
Piperacillin/tazobactam	≥128/R	≥128/R	≤4/S
**Cephalosporins**			
Cefazolin	≥64/R	≥64/R	≤4/S
Cefuroxime sodium	≥64/R	≥64/R	4/S
Cefuroxime axetil	≥64/R	≥64/R	4/S
Cefotetan	≥64/R	32/R	≤4/S
Ceftriaxone	≥64/R	≥64/R	≤1/S
Ceftazidime	≥64/R	≥64/R	≤1/S
**Carbapenems**			
Imipenem	≥16/R	≥16/R	≤1/S
Meropenem	≥16/R	8/R	≤0.25/S
**Monobactam**			
Aztreonam	≥64/R	≥64/R	≤1/S
**Fluoroquinolones**			
Ciprofloxacin	≥4/R	≤0.25/S	≤0.25/S
Levofloxacin	≥8/R	≤0.25/S	≤0.25/S
**Sulfonamide**			
Trimethoprim/sulfamethoxazole	≥320/R	≤20/S	≤20/S
**Aminoglycosides**			
Amikacin	≤2/S	≤2/S	≤2/S
Gentamicin	≤1/S	≤1/S	≤1/S
Tobramycin	2/S	≤1/S	≤1/S

*S, susceptible; R, resistant.*

**FIGURE 1:**
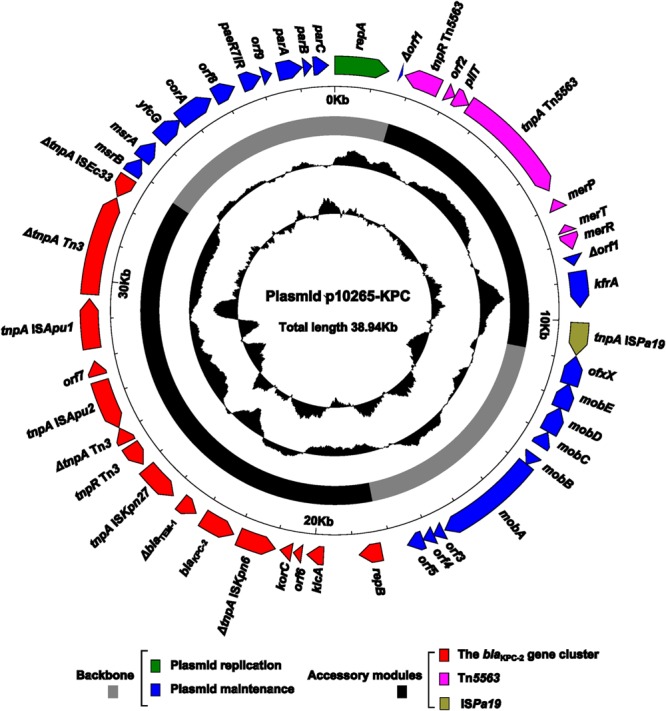
**Schematic maps of p10265-KPC.** Genes are denoted by arrows and colored based on gene function classification. The innermost two circles indicate the GC-Skew [(G-C)/(G + C)] and the GC content.

The complete sequence of p10265-KPC, recovered from the 10265-KPC-TOP10 strain, was determined with a mean coverage of 124, resulting in a circular plasmid sequence of 38,939 bp with an average G + C content of 58.2% (**Figure 1**). Sequence annotation generated a total of 41 predicted open reading frames. The modular structure of p10265-KPC is divided into the backbone [especially including the regions for plasmid replication (*repA*) and stability (*parABC* and *mob*)], and three separate accessory modules (a novel *bla*_KPC-2_ gene cluster, Tn*5563*, and IS*Pa19*) inserted at the different sites of the backbone.

### Accessory Modules of p10265-KPC

The rapid spread of *bla*_KPC_ genes in European and American countries is linked to their location in a Tn*3*-family unit transposon Tn*4401* with at least eight designated isoforms *a* to *g* and a separate *d* (Chen et al., 2012; Bryant et al., 2013; Chmelnitsky et al., 2014). Tn*4401b* is the prototype one and has a modular structure *tnpRA* (transposition core module)-IS*Kpn7*-*bla*_KPC_-IS*Kpn6*, which is delimited by two 39 bp inverted repeats (IRs), and the other isoforms result from distinct deletion or insertion events occurring at the different sites of Tn*4401b* (Chen et al., 2012; Bryant et al., 2013; Chmelnitsky et al., 2014).

**FIGURE 2:**
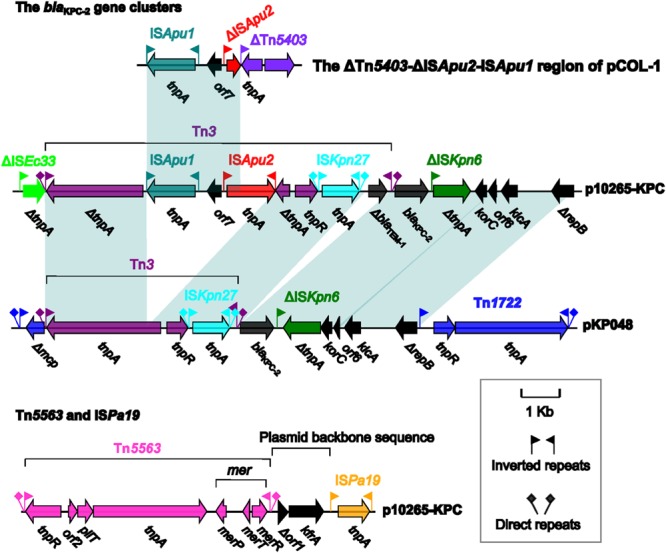
**Accessory modules of p10265-KPC and comparison with related genetic contents.** Genes are denoted by arrows and colored based on gene function classification. Shading regions denote shared regions of homology (>95% nucleotide similarity).

In China, Tn*4401* is rarely found (Ho et al., 2013) and, instead, a core module Tn*3*-IS*Kpn27*-*bla*_KPC_-ΔIS*Kpn6* is frequently identified as the *bla*_KPC_ platform (Shen et al., 2009; Chen et al., 2014a,c; Li et al., 2015). For generation of the above core module, an intact IS*Kpn27* is inserted into the 3′-end of Tn*3*, and then the resulting Tn*3-*IS*Kpn27* is connected with *bla*_KPC-2_ and ΔIS*Kpn6* (**Figure 2**). This core module is imbedded in two major classes of transposons (Wang et al., 2015), the Tn*1722*-based unit transposons [e.g., pKP048 (Shen et al., 2009)] and the IS*26*-based composite transposons [e.g., pKPC-LKEc (Chen et al., 2014c)].

The Tn*1722*-based transposon in pKP048 (Shen et al., 2009) looks like a prototype one, and it is generated from the insertion of IS*Kpn27-bla*_KPC-2_*-*ΔIS*Kpn6-korC-orf6*- *klcA-*Δ*repB* into the *mcp* gene of the cryptic transposon Tn*1722*, making *mcp* to be truncated; moreover, it is bounded by 39 bp IRs and further flanked by 5 bp direct repeats (DRs: target site duplications which are usually the signature of a transposition event) at both ends (**Figure 2**). Before being captured by Tn*1722*, the above mentioned core module IS*Kpn27-bla*_KPC-2_*-*ΔIS*Kpn6* is connected with a gene cluster *korC-orf6*-*klcA-*Δ*repB*.

In p10265-KPC, the primary genetic content Tn*3-*IS*Kpn27-bla*_KPC-2_*-*ΔIS*Kpn6 -korC-orf6*-*klcA-*Δ*repB* is also found but it has undergone two major evolutionary events: (i) the insertion of a Δ*bla*_TEM-1_ gene between IS*Kpn27* and the Tn*3* IRR (IR right), and (ii) the insertion of IS*Apu1*-*orf7*-IS*Apu2* into the Tn*3 tnpA* gene, disrupting it into two separate parts (**Figure 2**). The resulting structure is then connected with ΔIS*Ec33* to finally constitute a 15.1 kb ΔIS*Ec33*-associated element ΔIS*Ec33*-Tn*3*-IS*Apu1*-*orf7*-IS*Apu2*-IS*Kpn27-*Δ*bla*_TEM-1_*-bla*_KPC-2_*-*ΔIS*Kpn6-korC- orf6*-*klcA-*Δ*repB* (**Figure 2**). In contrast to the Tn*1722*-based unit transposons, the ΔIS*Ec33*-associated element is not bracketed by IRs and DRs. In addition, the Tn*3* in this ΔIS*Ec33*-associated element is heavily fragmented due to insertion of various elements and most likely defective in the activity of transposition. The mobilization of this ΔIS*Ec33*-associated element in p10265-KPC would attribute to homologous recombination-based insertion of a foreign element Tn*3-*IS*Apu1*-*orf7*-IS*Apu2*-IS*Kpn27-*Δ*bla*_TEM-1_*-bla*_KPC-2_*-*ΔIS*Kpn6-korC-orf6*-*klcA-*Δ*repB* into a pre-existent intact IS*Ec33* element (making it truncated at 3′ end), rather than resulting from a transposition event of the whole ΔIS*Ec33*-associated element followed by the deletion of its adjacent extremities removing IR and DR sequences.

In p10265-KPC, the 6.5 kb transposon Tn*5563* is located upstream of IS*Pa19*, with two consecutive backbone genes *orf6* and *kfrA* as the interval between Tn*5563* and IS*Pa19*. IS*Pa19* contains the single transposase gene *tnpA*, and this insertion sequence is bordered by 18 bp IRs; this insertion sequence was initially described in plasmid Rms149 (Haines et al., 2005). Tn*5563*, belonging to the Tn*3* subgroup of the Tn*3* family transposons, was initially identified in plasmid pRA2 from *P. alcaligenes* and organized sequentially as *tnpR* (resolvase), *orf2* (hypothetical protein), *pliT* (PilT domain-containing protein), *tnpA, merP* (mercuric transport protein periplasmic componen), *merT* (mercuric transport protein), and *merR* (mercuric resistance operon regulatory protein); this gene cluster is bounded by 39 bp IRs and further flanked by 5 bp (this study) or 7 bp DRs at both ends (Yeo et al., 1998). Various truncated versions of Tn*5563* are also found in pOZ176 (Xiong et al., 2013) and pUM505 (Ramirez-Diaz et al., 2011) from *P. aeruginosa*. The Tn*5563* element of p10265-KPC differs from the prototype Tn*5563* of pRA2 with a 286 bp insertion (nucleotide position 7270 to 7555) between *merP* and *merT*. The above observations indicate the frequent inter-plasmid transmission of Tn*5563* among *Pseudomonas* species.

### Replication and Maintenance Determinants of p10265-KPC

Plasmid p10265-KPC belongs to the IncP-6 incompatibility group because it carries three partition genes *parABC* and a downstream replicase gene *repA*, which constitute an IncP6-type consecutive *par-rep* gene cluster. The *parA* gene encodes an ATPase, whereas *parB* and *parC* encode auxiliary partition proteins. The *repA* gene and the *parABC* locus of p10265-KPC show >97% and >99%, respectively, nucleotide sequence identity with the three IncP-6 plasmids Rms149, pRIO-5, and pCOL-1. The Rms149 RepA has been shown to confer the plasmid’s replication ability in *E. coli, P. aeruginosa*, and *P. putida* (Haines et al., 2005), while the pRIO-5 RepA is able to replicate in *S. marcescens* and *Acinetobacter baumannii* but not in *P. aeruginosa* (Bonnin et al., 2012). The *parABC* locus of Rms149 is known to promote the plasmid mobilization in *E. coli* (Haines et al., 2005).

All of p10265-KPC, Rms149, pRIO-5, and pCOL-1 contain a 5.6 kb MOB_P_ family mobilization module (Francia et al., 2004), which is composed of six genes *mobA* (relaxase/primase fusion protein), *mobB* (*oriT* recognition-like protein), *mobC* (relaxosome protein), and *mobD* and *mobE* (auxiliary proteins). The study with Rms149 denotes that the *mob* gene cluster is functional for the plasmid mobilization in *E. coli* (Haines et al., 2005). In addition, the above *mob* gene clusters are similar to those of the IncQ plasmids pTF-FC2 and pTC-F14; as shown by the genetic analysis using these two plasmids, the minimal region essential for mobilization are *mobA, mobB*, and *mobC*, while *mobD* and *mobE* are non-essential but together they greatly enhance the mobilization frequency (van Zyl et al., 2003).

### Genomic Comparison of p10265-KPC with pCOL-1

**FIGURE 3:**
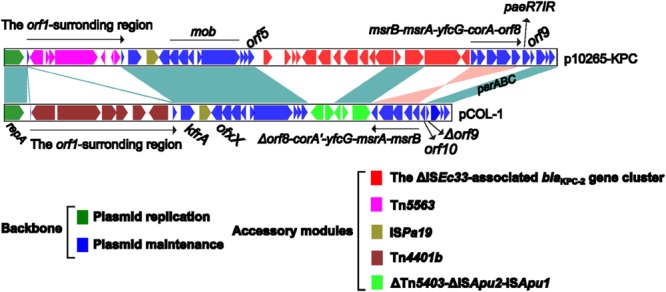
**Linear comparison of sequenced plasmids p10265-KPC and pCOL-1.** Genes are denoted by arrows and colored based on gene function classification. Shading regions denote shared regions of homology (>95% nucleotide similarity).

pCOL-1 represents the first sequenced KPC-producing IncP-6 plasmid (Naas et al., 2013), while p10265-KPC is the second one. The *par*-*rep* regions for partition-replication and the *mob* gene modules for mobilization are conserved in the p10265-KPC and pCOL-1 backbones (**Figure 3**), allowing these two plasmids to transfer if a conjugative plasmid is also present in the cell. The p10265-KPC and pCOL-1 backbones lack the conjugal transfer gene regions, which is in accordance with the fact that these two plasmids are unable to self-transfer their drug resistance markers via conjugation. An inversion of the backbone gene cluster *msrB-msrA-yfcG-corA-orf8* in p10265-KPC turns it into Δ*orf8-corA′-yfcG-msrA-msrB* in pCOL-1 with a truncation of *orf8* and, moreover, *corA* becomes a pseudo gene with the accumulation of multiple indels.

**FIGURE 4:**
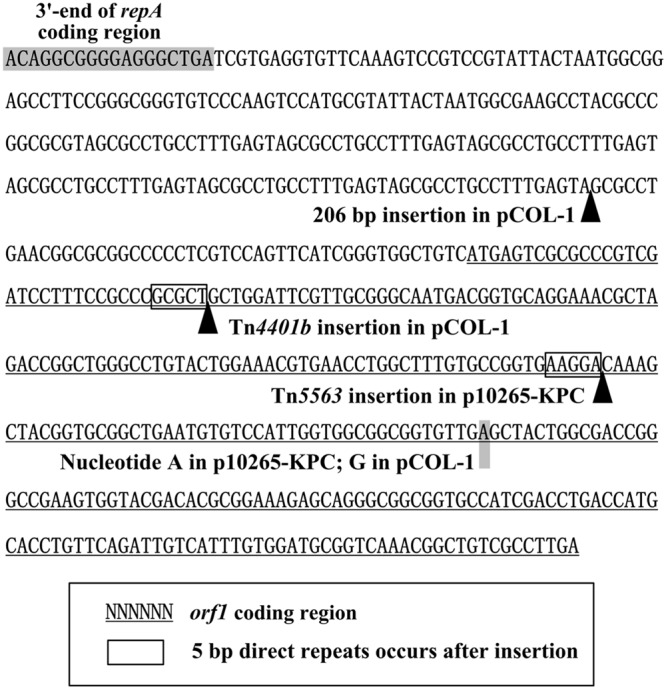
***orf1*-surrounding regions.** Shown are the *orf1*-surrounding DNA regions of p10265-KPC and pCOL-1 with inserted genetic contents.

A 206 bp fragment comprising 12 copies of tandem repeat of GCGCCTGCCTTTGAGTA is inserted into the *repA-orf1* intergenic region of pCOL-1 relative to p10265-KPC (**Figures 3** and **4**). The open reading frame *orf1* is disrupted at two distinct sites, respectively, by the insertion of two different accessory elements, namely a 10 kb *bla*_KPC-2_-carrying Tn*4401b* in pCOL-1 and a *mer* locus-carrying *Tn5563* in p10265-KPC (**Figures 3** and **4**).

pCOL-1 and p10265-KPC share the accessory element IS*Pa19*, which is inserted between the backbone genes *kfrA* and *ofxX* (**Figure 3**). Downstream of the backbone gene *orf5* of p10265-KPC and pCOL-1 are two distinct inserted accessory regions, namely the ΔIS*Ec33*-associated *bla*_KPC-2_ gene cluster and a 4.3 kb ΔTn*5403*-ΔIS*Apu2*-IS*Apu1* region (showing sequence similarity to the ΔIS*Ec33*-associated element), respectively (**Figures 2** and **3**).

Remarkably, pCOL-1 and p10265-KPC carry two distinct *bla*_KPC-2_ gene clusters, Tn*4401b* and the ΔIS*Ec33*-associated element, respectively; these two gene clusters are inserted at two different sites of the IncP-6 backbone and seem to have completely different evolutionary histories of genetic assembly and transposition.

## Conclusion

Plasmid p10265-KPC is a novel IncP-6 resistance plasmid from *P. aeruginosa*, and it carries the IncP-6-type replication, partition and mobilization systems and a novel ΔIS*Ec33*-associated *bla*_KPC-2_ gene cluster accounting for carbapenem resistance. The ΔIS*Ec33*-associated element has a complex mosaic structure, which is genetically related to the previously characterized Tn*1722*-based *bla*_KPC-2_-carrring unit transposons (Wang et al., 2015). Besides *bla*_KPC-2_, p10265-KPC still carries a truncated *bla*_TEM-1_ gene in the *bla*_KPC-2_ gene cluster and an incomplete *mer* locus in Tn*5563*, both of which are thought defective to mediate the corresponding resistance phenotypes. KPC-2 appears to be the sole determinant of antimicrobial resistance for p10265-KPC. Epidemiological study and routine surveillance of p10265-KPC-like plasmids in China is needed.

The IncP-6 resistance plasmids are not commonly found in the natural isolates of *P. aeruginosa*. The accumulating complete sequences of the IncP-6 plasmids would make it possible to chart their evolutionary history and to draw the inferences about the processes that lead to these complex plasmid genomes. As seen from p10265-KPC, Rms149, pRIO-5, and pCOL-1, the IncP-6 plasmid backbones are able to integrate a wide variety of foreign genetic contents through transposition or homologous recombination mediated by the transposable elements such as insertion sequences, transposons and integrons. The IncP-6 plasmids are quite unusual in having a relatively small backbone but carrying a large amount of accessory modules which are mainly composed of mobile genetic elements and resistance determinants. It is worth elucidating whether there are specific mechanisms associated with the IncP-6 plasmids to promote the involvement of themselves in the complex processes of acquisition of foreign genetic contents.

## Author Contributions

DZ, FS, and XZ designed the study. XD, WX, JF, WL, GL, HW, and FS performed experiments. DZ, XD, and FS analyzed data. XD, WX, JF, WL, GL, HW, and FS contributed reagents, materials and analysis tools. DZ, XD, FS, and XZ wrote this manuscript.

## Conflict of Interest Statement

The authors declare that the research was conducted in the absence of any commercial or financial relationships that could be construed as a potential conflict of interest.

## Footnotes

1 http://www.lahey.org/studies/

2 https://inkscape.org/
